# Odor quality profile is partially influenced by verbal cues

**DOI:** 10.1371/journal.pone.0226385

**Published:** 2019-12-12

**Authors:** Jisub Bae, Ju-Yeon Yi, Cheil Moon

**Affiliations:** 1 Department of Brain and Cognitive Sciences, Daegu Gyeongbuk Institute of Science and Technology, Daegu, Republic of Korea; 2 Convergence Research Advanced Centre for Olfaction, Daegu Gyeongbuk Institute of Science and Technology, Daegu, Republic of Korea; Monell Chemical Senses Center, UNITED STATES

## Abstract

Characterizing an odor quality is difficult for humans. Ever-increasing physiological and behavioral studies have characterized odor quality and demonstrated high performance of human odor categorization. However, there are no precise methods for measuring the multidimensional axis of an odor quality. Furthermore, it can be altered by individual experience, even when using existing measurement methods for the multidimensional axis of odor such as odor profiling. It is, therefore, necessary to characterize patterns of odor quality with odor profiling and observe alterations in odor profiles under the influence of subjective rating conditions such as verbal cues. Considering the high performance of human odor categorization, we hypothesized that odor may have specific odor quality that is scarcely altered by verbal cues. We assessed odor responses to isovaleric acid with and without verbal cues and compared the results in each stimulation condition. We found that verbal cues influenced the rating of odor quality descriptors. Verbal cues weakly influenced the odor quality descriptors of high-rated value (upper 25%) compared to odor quality descriptors of low-rated value (lower 75%) by the survey test. Even under different verbal cue conditions, the same odor was classified in the same class when using high-rated odor quality descriptors. Our study suggests that people extract essential odor quality descriptors that represent the odor itself in order to efficiently quantify odor quality.

## Introduction

Characterizing odor quality is important as humans can quantify differences between smells in order to predict odor quality. Due to its importance, numerous studies have provided evidence for quantifying odor quality. From a physiological viewpoint, perceiving odor begins with the activation of a specific odorant receptor (OR) repertoire in response to volatile chemicals [1–3]. This odor information processes and categorizes each specific odor quality in the brain [4–6]. Studies suggest that detected odors are processed into specific information that relate with the quality of odor perception. Moreover, categorizing odor and identifying odor is coded in different regions of the brain [7, 8]. Physiological evidence suggests that odor quality can be influenced by survey or behavioral output, and supports the possibility that odor quality can be quantified. Increasing evidence of high-performance odor discriminatory ability by humans suggests that humans possess high performance in categorizing odors despite possessing low performance in identifying those odors [9–12].

Despite the evidence, quantifying odor quality is a well-known issue. One of the reasons is that odor perception has a multidimensional axis with less evidence for perceptual space [13, 14]. Although physiological evidence suggests the existence of precise abilities for odor quantification, more evidence is required in order to predict the multidimensional axis of odor perception. Measuring odor discrimination is also rife with defects for quantifying odor. As the discriminating odor task does not represent the multidimensional axis of odor, there are no prior limits on describing odor differences. Based on these observations, odor profiling is the correct quantification method as it measures the multidimensional axis of odor by rating various descriptors [15, 16].

Profiling odors is challenging as it may occur by the subjective rating of odor quality descriptors. As odor responses can differ based on individual experience, odor profile can also differ by condition, despite being stimulated with the same odor. For instance, odor responses vary by individual experiences [17–20]. Training and experience increase the discrimination accuracy of the components in odor mixtures [17], and cultural differences lead to different responses to odor categorization [18, 21, 22]. Moreover, presenting verbal cues with odor stimulation alters odor responses, suggesting that odor perception is significantly influenced by verbal labeling [19]. These studies suggest that subjective rating can be influenced by individual experience and that measuring odor profiles is less accurate than other methods involving behavioral or neurological approaches [5, 9].

It is, therefore, necessary to characterize alterations of odor profiles stimulating odor by influencing subjective rating conditions. We hypothesized that odor may have a specific primary odor quality which is barely altered by subjective rating conditions. Although odor responses can be altered in some way with respect to odor quality factors under verbal cue conditions, invariant or fewer variant parts of odor profiling may exist [7]. To address this hypothesis, we characterized altering odor quality patterns in response to the same odor under verbal cues as previous research suggests that verbal cues can influence odor quality perception [19, 23–26]. For instance, perceived intensity of food odors increased when presented in colored liquid [23], and fragrance pleasantness and sweetness perception were altered when supplied with or without brand labels [24]. Furthermore, odor response was significantly influenced by different verbal cues when odors were presented with either positive or negative cues [19, 25]. Therefore, we measured odor quality by profiling methods while presenting different verbal cues to observe the altering patterns of odor quality. We used isovaleric acid (IVA) and heptanol (Hep) for this purpose. We assessed odor responses to IVA with and without verbal cues, and Hep was also assessed. As IVA is known as an ambiguous odor that can induce significantly different responses in the brain [27], it is an appropriate model for characterizing alterations of odor profiles. Hep is used as a negative control due to its completely different odor quality compared to IVA. Since we measured alterations of IVA odor profiles by verbal cues, Hep was used as the standard for measuring the range of alteration. Although it is hard to understand how severely the odor profiles are altered by conditions, people can predict the amount of severity using negative controls such as Hep.

## Materials and methods

### Experimental scheme

Participants took part in groups (see Participants, Material and methods) for experiments at different times. Other experimental conditions were standardized (e.g., location, temperature, humidity, and illuminance). Each group of participants was tested in a room where the temperature and ventilation of odor were controlled. Room temperature was set to 21°C to ensure a fixed vapor pressure of the odor. Prior to the experiments, prepared odor and survey sheets were given to each participant as well as the instructions for the experimental procedure.

**Fig 1:**
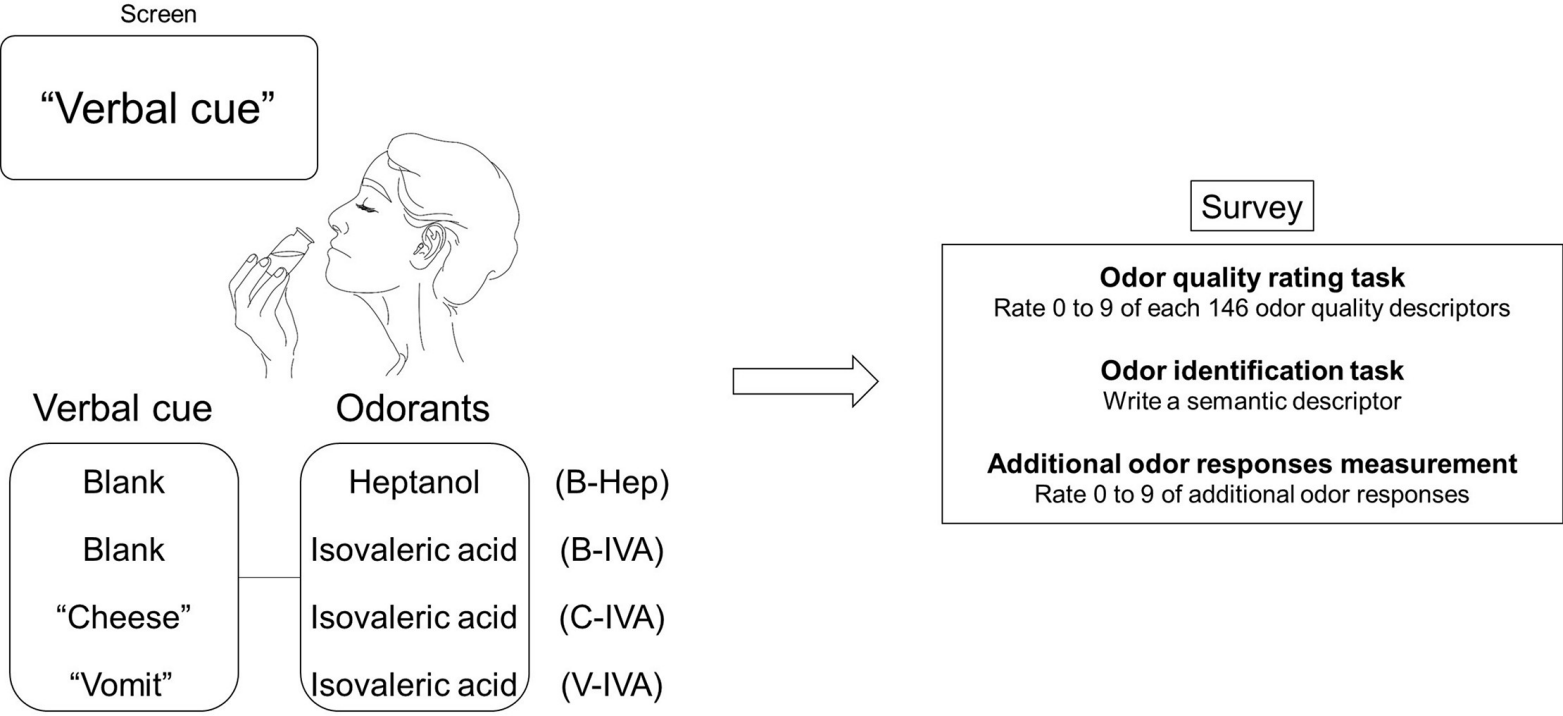
Experimental scheme. Odor stimulations are given while presenting verbal cues to the screen. Blank-Heptanol (B-Hep), blank-isovaleric acid (B-IVA), cheese-isovaleric acid (C-IVA) and vomit-isovaleric acid (V-IVA) are stimulation conditions. The survey was performed by presenting stimulations.

The experiment began with the odor quality-rating test of a verbal cue-odor stimulation (Fig 1). Odor stimulations were given while presenting verbal cues on the screen. For group 1, IVA and Hep with a blank screen (B-IVA, B-Hep) were presented at different times. For group 2, IVA with the “Cheese” verbal cue (C-IVA) was presented. For group 3, IVA with the “Vomit” verbal cue (V-IVA) was presented. A total of 146 descriptors on odor quality were given in order to evaluate odor qualities (S1 Table), and six questionnaires were provided to evaluate additional odor responses; including identifying the odor, pleasantness, intensity, familiarity, edibility, and the relaxing effect (S2 Table). Discussion among the participants was not allowed until the end of the experiment.

About one month later, we conducted the second experiment. The experimental scheme was similar to the first experiment except that we performed each experimental condition with a different group. For group 1, IVA with the “Cheese” verbal cue (C-IVA) was presented. For group 2, IVA with the “Vomit” verbal cue (V-IVA) was presented. For group 3, IVA and Hep with the blank screen (B-IVA, B-Hep) were presented at a different time.

### Odorant preparation and delivery

Isovaleric acid (CAS number: 503-74-2, Aldrich, LOT#STBG4549V) and Heptanol (CAS number: 111-70-6, Aldrich, LOT#STBD9537V) were used as two odors. Isovaleric acid was diluted in mineral oil (CAS number: 8042-47-5, Sigma Aldrich, LOT#MKBZ6778V) to a 0.01% final concentration. Heptanol was also diluted in mineral oil to a final concentration of 0.5%. Odors were delivered using blotting paper. Two hundred microliters of the diluted odor was used to treat the tip of blotting paper and these prepared blotting papers were sealed in 50 ml falcon tubes. Odors were stimulated during experiments by unsealing the falcon tubes. Participants were allowed to continue sniffing the odorant with over a 30 sec interval between each sniff until they rated all the 146 odor quality descriptors.

### Participants

After providing informed consent, 96 students participated in this experiment (32 females, 64 males; mean age 21.02 years [SD = 2.54]). All participants displayed normal olfactory function and had no history of psychological or neurological diseases. Olfactory functions were examined using the Sniffin’ sticks, which quantity odor threshold and discriminatory ability [28]. Participants were divided into three groups randomly (Table 1). Group 1 (n = 32, 14 females, 18 males) was subjected to stimulation by IVA and Hep with the blank screen. Group 2 (n = 32, 10 females, 22 males) was subjected to stimulation by IVA with the “Cheese” verbal cue on the screen. Group 3 (n = 32, 8 females, 24 males) was subjected to stimulation by IVA with the “Vomit” verbal cue on the screen. Divided groups determined to be not significantly different with respect to age, odor threshold, or odor discrimination by one-way ANOVA. The study was approved by an Institutional Review Board ethics committee, Daegu Gyeongbuk Institute of Science & Technology (DGIST_170614-HR-012-01). For the second experiment, participants were recruited again about one month later. Group 1 (n = 16, 4 females, 12 males) was subjected to stimulation by IVA with the “Cheese” verbal cue on the screen. Group 2 (n = 16, 5 females, 11 males) was subjected to stimulation by IVA with the “Vomit” verbal cue on the screen. Group 3 (n = 16, 7 females, 9 males) was subjected to stimulation by IVA and Hep with the blank screen.

**Table 1:** Information on odor ability in each participants group (mean ± SD).

	Age	Threshold	Discrimination
**Group 1** **(B-IVA, B-Hep)**	21.00±2.83	6.47±2.02	10.28±1.05
**Group 2** **(C-IVA)**	21.31±3.00	5.97±1.99	10.16±1.14
**Group 3** **(V-IVA)**	20.75±1.55	5.94±2.00	10.25±1.16
**Statistics (p value)**	0.68 (ns)	0.49 (ns)	0.90 (ns)

Participants' information of age, odor threshold, and odor discrimination by each divided group. One-way ANOVA was performed for statistical comparison.

### Statistics

Results are shown as the mean ± standard error of the mean (SEM), and the significance threshold was set at p < 0.05. Two-way ANOVA was used to verify the survey rates of odor response. Bonferroni post hoc test was used to verify column differences. To verify correlations between the factors, Pearson’s linear correlation coefficient was used to calculate r values of each of the verbal cue-odor conditions. Principal component analysis (PCA) was used for multivariate analysis. Agglomerative hierarchical clustering (AHC) was used to verify odor quality similarity.

MATLAB 2016b and R were used for statistical analysis. Graphs displayed were created using GraphPad Prism 5.

### Survey

To measure the odor quality response, we evaluated 146 odor descriptions using a 1 to 9 rating scale (S1 Table), while 0 was used for an ‘Unknown description’. For the odor quality description task, participants chose the suitable scale of odor quality descriptions from 146 odor descriptions [29] following odor stimulation. To measure additional odor responses, we evaluated pleasantness, intensity, familiarity, edibility, and the relaxing effect using the 1 to 9 rating scales and recorded the odor description in order to define how participants identified odors (S2 Table).

### Verbal cues

“Cheese” and “Vomit” were used as verbal cues for IVA. These cues were selected from a previous study that illustrated different odor responses induced by different verbal cues [19]. Verbal cues were displayed on the projection screen with white letters on a black background. The blank condition was a black background without any letters.

## Results

### Verbal cues alter odor quality pattern

**Fig 2:**
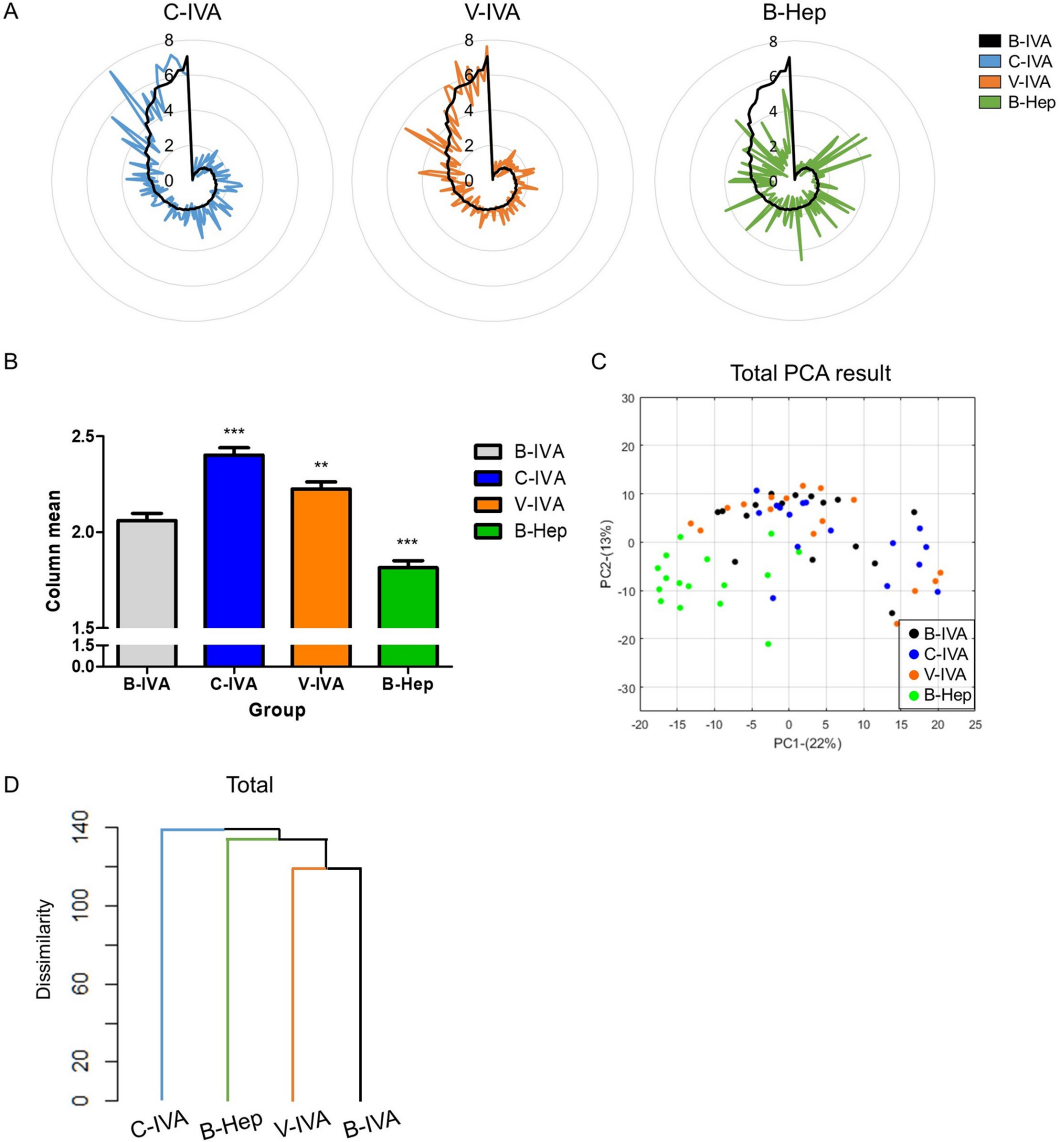
Patterns of odor descriptors among the different stimulation conditions. (A) Descriptor survey rates of each stimulation condition. A total of 146 values are presented as a radar chart in counter clockwise-descending ordered by B-IVA. The Black line is B-IVA, blue is C-IVA, orange is V-IVA, and green is B-Hep. (B) Verification of differences by stimulation conditions. By using two-way ANOVA, C-IVA, V-IVA, and B-Hep are significantly different from B-IVA. Two cued IVA conditions (C-IVA, V-IVA) were also significantly different from each other. (C) Odor quality space comprised principal component 1 (PC1: 22%) and principal component 2 (PC2: 14%). Each dot was projected from each participant’s 146 descriptor values. (D) Verification of similarity between stimulation conditions. Compared to B-IVA, V-IVA was next to B-IVA (161.50 distance), but C-IVA was rated as a more dissimilar distance (176.59 distance). B-Hep was rated as the most dissimilar condition compared to B-IVA (186.67 distance).

We first established that each stimulation condition exhibited different odor quality patterns by odor profiling, similar to previous studies [19, 23–26]. We performed odor descriptor survey tasks of B-IVA (verbal cue: blank, odor: IVA), C-IVA (verbal cue: “cheese,” odor: IVA), and V-IVA (verbal cue: “vomit,” odor: IVA) conditions in order to examine the effect of verbal cues. The B-Hep (verbal cue: blank, odor Hep) condition was used to define the standard for the severity of odor quality pattern alteration as Hep is a completely different smell from IVA (Fig 2A). To verify the overall influence of each condition, we compared the IVA conditions to B-Hep using two-way ANOVA. Each rating value of descriptors was used as a row factor and each condition was used as a column factor. We found significant differences between B-IVA, V-IVA, C-IVA, and B-Hep (F[3,18104] = 67.84, p < 0.0001, pη2 = 0.011, Two-way ANOVA) (Fig 2B). C-IVA and V-IVA were significantly higher than B-IVA in the survey rating values although these conditions used the same odor stimulation (p < 0.0001, Bonferroni post hoc test each). C-IVA was also significantly different from V-IVA (p < 0.0001, Bonferroni post hoc test). B-Hep was the second condition to be different from B-IVA. To verify the distance between conditions in odor quality space, the multivariate analysis, PCA, was performed. Using the multivariate analysis, odor quality pattern showed that C-IVA had similar distances as B-Hep from B-IVA. Specifically, spread patterns in odor quality space showed that components of C-IVA were located away from the center of B-IVA (Fig 2C). Although the density of elements was lower than B-Hep, centroid of C-IVA was spread in a similar distance pattern to B-Hep. In contrast, the spread pattern of V-IVA was similar to B-IVA. Finally, we performed a cluster analysis between the conditions (Fig 2D) to evaluate the distance. B-Hep was rated the most different class compared to B-IVA (186.67 distance), and C-IVA was rated at a similar distance from B-IVA (176.59 distance). V-IVA was rated in the same class as B-IVA (161.50 distance). To verify alterations in odor identification and pleasantness, additional survey tasks were performed. We found that C-IVA had significantly different identification and pleasantness patterns from B-IVA, although V-IVA was similar to B-IVA (S1 Fig, S1 and S3 Tables). Pleasantness was also shown to be significantly different between C-IVA and V-IVA (T[62] = 2.82, p = 0.0065). These data suggest that verbal cues induce alterations of odor quality patterns in IVA.

### Odor quality pattern altered differently depending on the rating score of descriptors by conditions

**Fig 3:**
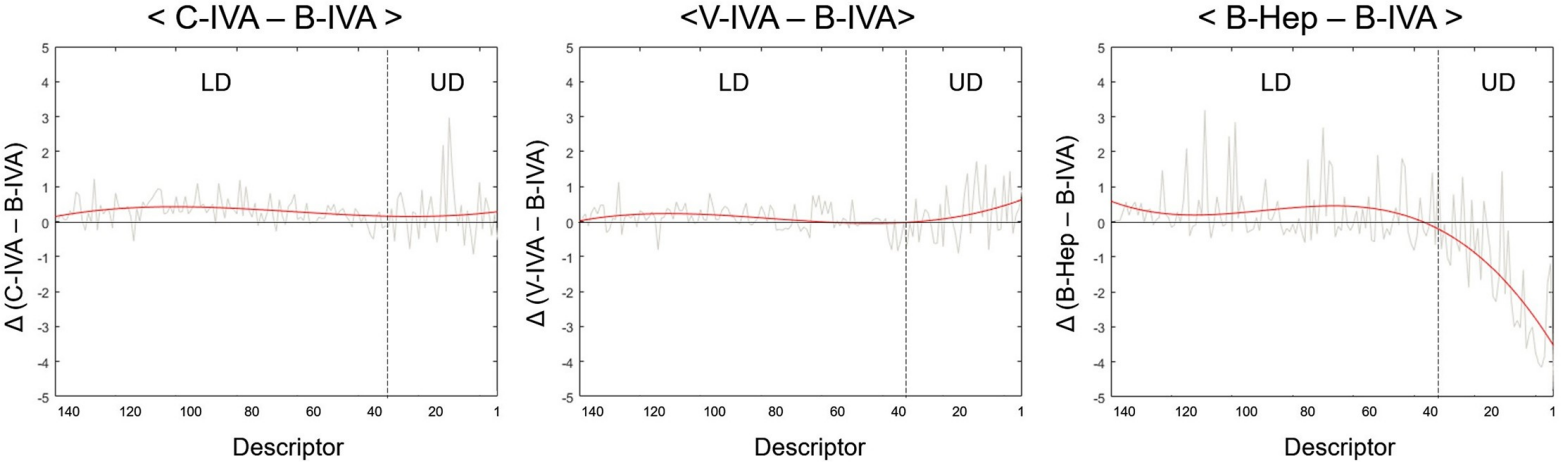
Different alteration patterns of odor descriptors depending on the rating score of the descriptor. Change of survey rate between B-IVA and other conditions. The X-axis represents descending order of descriptors from B-IVA odor quality rating (S4 Table). Y-axis represents the deviation of odor quality rating between each stimulation condition (C-IVA, V-IVA, B-IVA) and B-IVA condition. The gray line indicates changing values. The orange line indicates the curve fitting of the gray line. The dotted line indicates the boundary between upper 25% of data (UD) and the rest of the data (LD: lower 75% of data).

To examine the alterations, specifically in response to verbal cues and odors, we compared total descriptor ratings from stimulation conditions to B-IVA. We arranged descriptors in descending order with B-IVA rating values (S4 Table). Descriptors of other stimulation conditions were also arranged similar to B-IVA’s arrangement. Fig 3 shows the deviation between each stimulation condition and B-IVA. When comparing the rating score patterns of C-IVA, V-IVA, and B-Hep to B-IVA, we found that both C-IVA and V-IVA have similar patterns as B-IVA on total descriptors. However, B-Hep condition showed different patterns, especially in the upper 25% of data values. In contrast, the lower 75% of data values exhibited a smaller difference among conditions. These results suggest that upper 25% of data values represent odor quality descriptors more specifically to IVA rather than Hep. Based on these results, we defined ‘UD (upper 25% odor quality descriptors)’ as the upper 25% of B-IVA odor quality descriptors and ‘LD (lower 75% odor quality descriptors)’ as the lower 75% of B-IVA odor quality descriptors. Table 2 and Table 3 show the upper 25% odor quality descriptors of B-IVA and the upper 25% odor quality descriptors of C-IVA, V-IVA, and B-Hep, respectively.

**Table 2:** List of upper 25% odor quality descriptors in B-IVA.

1	sickening	16	sour	31	medicinal
2	aromatic	17	cheesy	32	wet wool, wet dog
3	stale	18	light	33	oily, fatty
4	dirty linen-like	19	sour milk	34	rubbery
5	rancid	20	cadaverous, like a dead animal	35	sulphidic
6	sweaty	21	cat-urine-like	36	fishy
7	putrid, foul, decayed	22	leather-like	37	cork-like
8	musty, earthy, moldy	23	warm	38	banana-like
9	like ammonia	24	chemical		
10	fecal (like manure)	25	mouse-like		
11	heavy	26	sharp, pungent, acid		
12	animal	27	mushroom-like		
13	sewer odor	28	bitter		
14	urine-like	29	yeasty		
15	fermented (rotten) fruit	30	rope-like		

List of upper 25% odor descriptors of B-IVA. This list defined as ‘UD’.

**Table 3:** List of upper 25% odor quality descriptors in C-IVA, V-IVA, and B-Hep.

			**<C-IVA>**		
1	aromatic	16	urine-like	31	fishy
2	putrid, foul, decayed	17	like ammonia	32	spicy
3	cheesy	18	animal	33	mouse-like
4	sickening	19	warm	34	buttery (fresh)
5	rancid	20	sharp, pungent, acid	35	cat-urine-like
6	dirty linen-like	21	cadaverous, like a dead animal	36	medicinal
7	sour milk	22	oily, fatty	37	soupy
8	stale	23	leather-like	38	beery (beer-like)
9	sweaty	24	light		
10	musty, earthy, moldy	25	rubbery		
11	sour	26	chemical		
12	sewer odor	27	yeasty		
13	heavy	28	bitter		
14	fecal (like manure)	29	wet wool, wet dog		
15	fermented (rotten) fruit	30	peanut butter		
			**<V-IVA>**		
1	sickening	16	urine-like	31	beery (beer-like)
2	rancid	17	cheesy	32	paint-like
3	aromatic	18	cadaverous, like a dead animal	33	stale tobacco smoke
4	putrid, foul, decayed	19	sharp, pungent, acid	34	rubbery
5	stale	20	animal	35	tar-like
6	sewer odor	21	bitter	36	burned rubber-like
7	fecal (like manure)	22	light	37	like gasoline, solvent
8	Sweaty	23	mouse-like	38	leather-like
9	sour	24	cat-urine-like		
10	dirty linen-like	25	fishy		
11	musty, earthy, moldy	26	chemical		
12	heavy	27	oily, fatty		
13	sour milk	28	wet wool, wet dog		
14	like ammonia	29	warm		
15	fermented (rotten) fruit	30	medicinal		
			**<B-Hep>**		
1	aromatic	16	orange (fruit)	31	herbal, green, cut grass
2	light	17	lavender	32	musty, earthy, moldy
3	fragrant	18	sour	33	tea-leaves-like
4	cool, cooling	19	bitter	34	wet paper-like
5	cologne	20	soapy	35	like gasoline, solvent
6	stale	21	rubbery	36	cork-like
7	medicinal	22	heavy	37	kerosene
8	perfumery	23	warm	38	burned rubber-like
9	like mothballs	24	sweet		
10	floral	25	grapefruit		
11	like cleaning fluid (carbona)	26	minty, peppermint		
12	fruity (other)	27	sharp, pungent, acid		
13	fruity (citrus)	28	chalky		
14	lemon (fruit)	29	paint-like		
15	chemical	30	leather-like		

List of upper 25% odor descriptors of C-IVA, V-IVA, and B-Hep. Grey colored cells represent descriptors which are not on the list of UD (Table 2: the upper 25% of B-IVA odor quality descriptors).

### UD descriptors are less altered compared to LD descriptors in IVA

**Fig 4:**
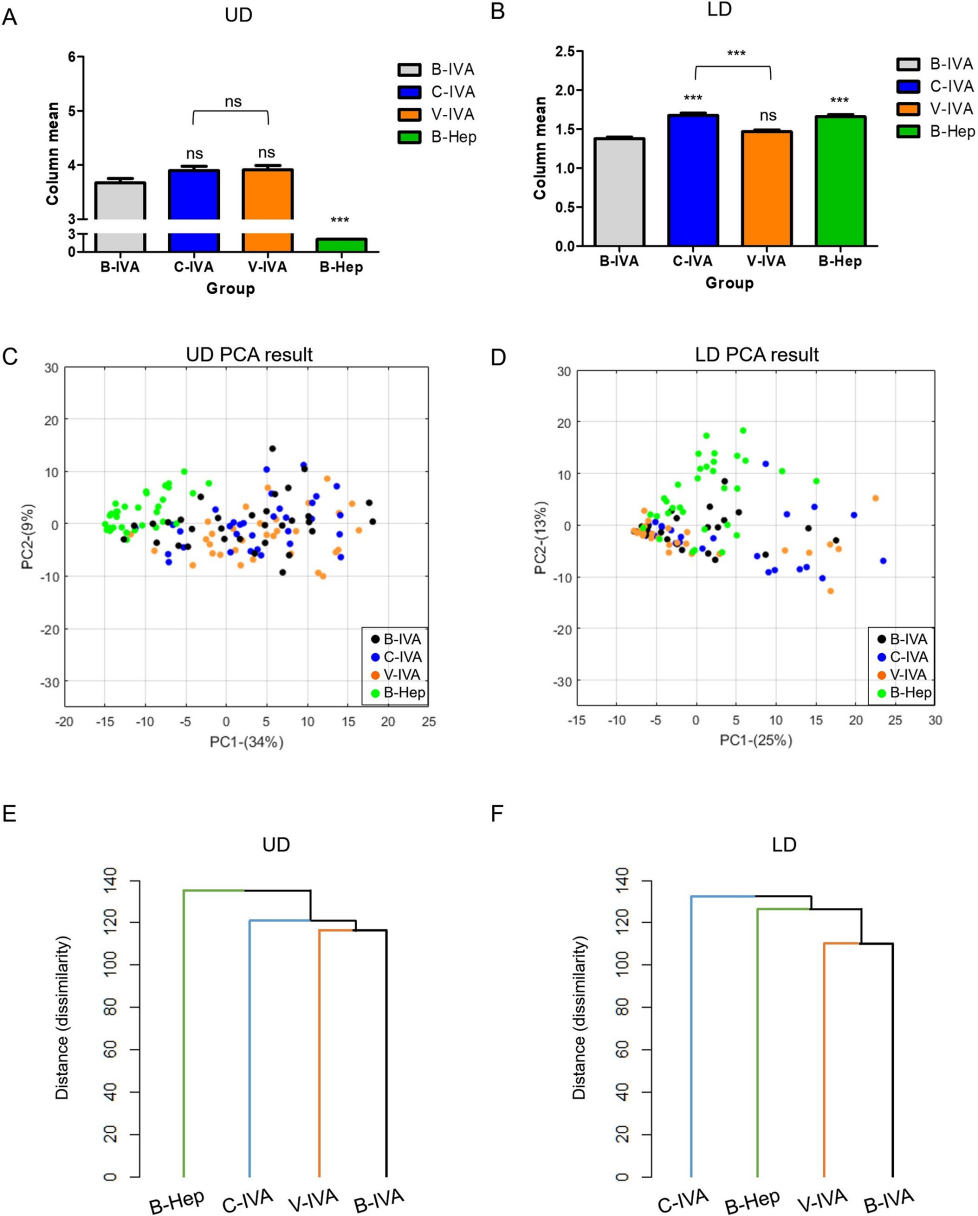
Different patterns of odor descriptors between two datasets separated by first quartile points. (A-B) Verification of differences in stimulation conditions. (A) By two-way ANOVA, B-Hep was significantly different from B-IVA but C-IVA and V-IVA had no differences. (B) By two-way ANOVA, C-IVA, V-IVA, and B-Hep were significantly different from B-IVA. (C-D) Odor quality space comprised PC1 (C: 34%, D: 25%) and PC2 (C: 9%, D: 13%). Each dot was projected from each participant’s 37 descriptor values in (C) and 109 descriptor values in (D). (E-F) Verification of similarity between stimulation conditions by cluster analysis. Y-axis represents dissimilarity. (E) Compared to B-IVA, V-IVA resided in the same class (116.27 distance) and C-IVA was the next closest class (121.25 distance). B-Hep is the most dissimilar condition (135.24 distance). (F) Compared to B-IVA, V-IVA resided in the same class (112.08 distance) and B-hep was the next closest class (125.78 distance). C-IVA is the most dissimilar condition (131.26 distance).

To verify differences between experimental conditions by UD and LD, we compared the differences of odor profiles between B-IVA, C-IVA, V-IVA, and B-Hep by separating UD and LD (Fig 4A and 4B, Table 4). In UD, we found no significant differences among the IVA conditions but observed significant differences when compared to B-Hep. Differences among the conditions were verified (F[3,4712] = 181.91, p < 0.0001, _p_η^2^ = 0.10, Two-way ANOVA), but post hoc test suggested that only B-Hep was rated significantly lower when compared to each IVA condition (p < 0.0001, Bonferroni post hoc test each). In LD, we found significant differences between the IVA conditions and the B-Hep condition. Differences among conditions were verified (F[3,13392] = 35.60, p < 0.0001, _p_η^2^ = 0.0079, Two-way ANOVA), and the post hoc test suggested that all conditions were significantly different from the B-IVA, except the V-IVA condition (B-IVA vs C-IVA, p < 0.0001 | B-IVA vs V-IVA, p = 0.061 | B-IVA vs B-Hep, p < 0.0001 | C-IVA vs V-IVA, p < 0.0001, Bonferroni post hoc test each). C-IVA and B-Hep rated significantly higher than B-IVA. These results demonstrate no significant differences between IVA conditions in UD, however, significantly reversed patterns were observed in LD. Moreover, B-Hep was significantly different from total IVA conditions in UD but not in different in LD.

**Table 4:** Comparison of each odor descriptor values between B-IVA and other stimulation conditions by t-test.

B-IVA vs C-IVA			B-IVA vs V-IVA			B-IVA vs B-Hep		
*O*.*D*.	*p-value*	*t-value*	*O*.*D*.	*p-value*	*t-value*	*O*.*D*.	*p-value*	*t-value*
**sour**	0.041	2.09	**sickening**	0.022	2.35	**sickening**	0.030	-10.78
**cheesy**	< 0.001	4.66	**rancid**	0.020	2.39	**aromatic**	< 0.001	-2.23
**sour milk**	0.002	3.23	**putrid, foul, decayed**	0.042	2.081	**stale**	< 0.001	-2.73
fragrant	0.034	2.17	**sewer odor**	0.019	2.41	**dirty linen-like**	0.043	-8.00
cinnamon	0.019	2.42	**sour**	0.018	2.44	**rancid**	0.025	-7.87
household gas	0.039	2.11	**sour milk**	0.048	2.015	**sweaty**	0.008	-8.56
peanut butter	0.008	2.72	cedarwood-like	0.035	-2.16	**putrid, foul, decayed**	< 0.001	-7.37
etherish, anesthetic	0.030	2.23	dry, powdery	0.007	2.79	**musty, earthy, moldy**	0.006	-5.20
buttery (fresh)	0.039	2.10	dill-like	0.014	2.54	**like ammonia**	0.035	-5.73
soupy	0.006	2.82				**fecal (like manure)**	0.001	-7.19
dry, powdery	0.002	3.31				**heavy**	0.014	-2.066
creosote	0.018	2.43				**animal**	< 0.001	-6.69
caramel	0.046	2.03				**sewer odor**	0.007	-5.17
popcorn	0.013	2.56				**urine-like**	0.002	-5.19
spicy	0.019	2.40				**fermented (rotten) fruit**	< 0.001	-4.46
						**cheesy**	< 0.001	-4.39
						**light**	< 0.001	2.30
						**sour milk**	< 0.001	-2.79
						**cadaverous, like a dead animal**	0.001	-4.40
						**cat-urine-like**	0.001	-3.38
						**mouse-like**	< 0.001	-3.67
						**mushroom-like**	0.034	-2.17
						**yeasty**	< 0.001	-3.36
						**medicinal**	0.002	2.16
						**sulphidic**	0.002	-2.76
						**fishy**	0.001	-3.30
						cool, cooling	< 0.001	5.21
						fragrant	0.007	7.36
						cologne	0.012	7.17
						fruity (citrus)	< 0.001	2.86
						hay	< 0.001	-2.15
						fruity (other)	< 0.001	2.53
						floral	0.035	2.81

Each stimulation conditions are compared with B-IVA odor descriptor values from the odor quality rating task. This table only shows significantly different odor descriptors (rest of odor descriptors are n.s.). Odor quality descriptors listed in UD (Table 2: the upper 25% of B-IVA odor quality descriptors) are indicated in bold characters. O.D indicates Odor descriptor. Two-tail t-test was performed for statistical analysis (degree of freedom DF = 62).

To verify alterations of the distance between stimulation conditions in the odor quality space, we conducted a multivariate analysis displaying spread patterns in the odor quality space. We found that dots of the IVA conditions in UD were uniformly distributed, whereas B-Hep condition dots were scattered to the left side of IVA conditions dots (Fig 4C). On the other hand, C-IVA dots in LD were scattered to the right side of B-IVA and V-IVA (Fig 4D). Clustering analysis exhibited precise alteration distances between the conditions. We found that UD appeared to represent IVA odor qualities much better than LD (Fig 4E and 4F). Specifically, IVA conditions were clustered in a similar class in UD (Fig 4E). B-IVA and V-IVA were clustered in the same class, and C-IVA was clustered in the next closest class. B-Hep was clustered in the most distant class in UD. In contrast, IVA clusters were dissembled in LD (Fig 4F). C-IVA was further away from V-IVA and B-IVA, even in greater distance than B-Hep which exhibited different odor quality profiles. These results suggest that UD represents a better consensus IVA odor quality than LD.

To verify verbal cue effects on LD, we conducted the second experiment using similar experiment schemes albeit with different groups. The rationale for the second experiment was to test the hypothesis that whether the first and second experiments may have high correlation results if the verbal cue effect on LD is more intense than the random variation effect on LD. If not, the two experiments will exhibit low or no correlation results. Sixteen participants of each group (total of 16 X 3 = 48 participants) participated in the second experiment. Participants of Group 1 (assigned to B-IVA, B-Hep conditions in the first experiments, Table 1), were assigned to C-IVA condition, participants of Group 2 (assigned to C-IVA condition in the first experiments, Table 1) were assigned to V-IVA condition, and participants of Group 3 (assigned to V-IVA condition in the first experiments, Table 1) were assigned to B-IVA and B-Hep conditions.

We first verified the statistical patterns of the second experiment’s results. We compared the differences of the odor profiles among B-IVA, C-IVA, V-IVA, and B-Hep by separating UD and LD as in the first experiment. In UD, we found no significant differences among the IVA conditions but observed significant differences compared to B-Hep (S2A Fig). In LD, we found significant differences between the IVA and B-Hep condition (S2B Fig). We also conducted the same multivariate analysis, PCA, to observe spread patterns in the odor quality space. We found that dots of IVA conditions in UD were uniformly distributed, whereas B-Hep condition dots were scattered to the left side of IVA conditions dots (S2C Fig). In LD, on the other hand, C-IVA dots were scattered to the right side of B-IVA and V-IVA (S2D Fig). Clustering analysis showed alteration distances between the conditions. We found that UD showed a more distant B-Hep from the IVA conditions compared to LD (S2E and S2F Fig). Specifically, IVA conditions in both UD and LD were clustered in a similar distance, but B-Hep was clustered in more distance in UD compared to LD. The second experiment results demonstrate no significant differences between IVA conditions in UD, however significantly reversed patterns were observed in LD, similar to the first experimental results. Despite the low effect in the clustering analysis, most of statistical patterns were similar to the first experiment.

We subsequently performed a correlation analysis to verify the precise similarities between the first and second experiments. We examined the similarity between the data of randomly selected 16 participants from the first experiments and the data of 16 participants from the second experiment. As the bias of data can affect correlation results, shuffled data of the first experiment was used as a negative control. Since we used the data of 16 randomly selected participants from the first experiment for correlation analysis, we first verified if the number of sampling trials was appropriate by evaluating SEM of correlation coefficient (r-value). We found that 50 trials were appropriate (S3 Fig). Based on these results, we performed a correlation analysis. We found high correlation results in both UD and LD (S4 Fig). In UD, most trials exhibited a 0.85 to 0.90 r-value (Sum of trial = 37). Moreover, LD most of the trials exhibited a 0.65 to 0.75 r-value (Sum of trial in 0.65 to 0.70 = 25, Sum of trial in 0.65 to 0.70 = 18). These results suggest that the first and second experiment results from independent participants highly correlate in both UD and LD.

## Discussion and conclusions

We found that the upper 25% of odor quality values (UD) were altered less significantly than the lower 75% of odor quality values (LD) in response to a verbal cue. These findings may suggest that high ranked odor quality is less affected by verbal cues. We first verified, from previous studies [30, 31], that verbal cues can induce alterations in odor responses. We found that verbal cues do indeed induce alterations in odor quality responses (Fig 2), as previously suggested [30, 31]. Alterations in odor identification and emotional responses also support the fact that verbal cues induced alterations of odor reactions (S1 Fig, S1 and S3 Tables), comparable to previous studies [19, 23–26]. Interestingly, the effect of the verbal cue did not affect overall odor quality values, but parts of odor quality values. As shown in Fig 3, completely different odor (B-Hep) displayed massively different patterns in the upper 25% of odor quality descriptors of IVA (B-IVA). As Hep is a completely different odor from IVA in terms of odor quality, upper 25% of odor quality descriptors may be more related to the IVA specific odor quality. Moreover, our study shows that verbal cues induce a much less alteration in high rated odor quality descriptors but strongly influence low rated odor quality descriptors (Fig 4). These results suggest that primary odor quality can be represented by over upper 25% of odor quality values, and verbal cue may have a weak influence on this primary odor quality.

We first found that verbal cues exerted a weak influence on the high rated odor quality values (Fig 4 and Table 4). According to previous studies, training and experience may alter odor responses [17]. Moreover, cultural differences influence odor categorization [18, 21] and individual differences in odor perception [20]. These findings suggest that rating odor quality is influenced by top-down modulation, including by verbal cues that make evaluating odor quality somewhat difficult. Similarly, our study suggests that verbal cues modulate odor quality responses, with even more distance values (Fig 2B) and a similar distance compared to different odors (Fig 2D). However, we also found that these influences may not alter the whole odor quality profile and may primarily only alter low ranked odor quality values (Fig 4). High rated descriptors were weakly affected and were more related to verbal cues compared to low rated descriptors. Although high rated descriptors were also affected by cues (Tables 3 and 4), these changes were not strongly affected in evaluating overall odor responses (Fig 4).

Weak alterations in UD indicate that a high rated odor quality represents the primary odor quality of the targeted odorant and these primary dimensions may be characterized by a subjective rating. From the molecular logic of smell, each chemical possesses a specific odor quality because each chemical activates a specific OR repertoire set [1–3]. Subsequent studies have suggested that the brain categorizes chemical differences [4–6]. Moreover, in spite of having a low performance on identifying and naming odors in humans [32, 33], increasing evidence of high performance on odor discrimination ability of humans suggests that humans may have sufficient ability to characterize each specific odor [9–12]. Similar to the previous studies, our findings show that humans may perceive specific odor quality. High rated descriptors were rarely affected by verbal cues (Fig 4A, 4C and 4E), although verbal cue can modulate parts of descriptors in high rated descriptors (Fig 2, S1 Fig, S1 Table, S3 Table). Our studies suggest that high rated descriptors may represent primary odor quality of the targeted odorant. Additionally, these findings imply that specific odor quality perception can be represented by a survey task of rating odor descriptors.

We also found that UD was affected by verbal cues, though the influence was weak. Tables 3 and 4 suggest that descriptors were changed by verbal cues, although they were rated enough to be categorized in UD. One possible explanation for these alterations in UD is that the range of UD (upper 25%) was not perfect for representing specific odor quality. This is because altered descriptors under cued conditions were rated lower than other UD descriptors (Table 3; C-IVA, V-IVA table). In the case of different odor conditions, entire odor descriptors were altered regardless of higher or lower ratings in UD (Table 3; B-Hep). These results suggest that higher rated UD descriptors may have a more appropriate representation of odor quality in our experimental conditions, implying that inherent odor quality may not be perfectly represented by UD. An additional possible explanation is that altered descriptors may be related to the verbal cues. Table 4 suggests that the ‘cheese’ cue condition (C-IVA) altered the ‘cheesy’ descriptors and other dairy descriptors (sour milk, sour). Moreover, ‘vomit’ cue condition (V-IVA) altered ‘sickening’, ‘rancid’ and ‘putrid, foul, and decayed’, which can be related to the word, ‘vomit’. These alterations occurred throughout the UD list, regardless of high or low rated descriptors. These results suggest that some descriptors can be altered in response to cues. Therefore, a particular descriptor may be altered by proper cues that were very relevant to the descriptor.

Some researchers recently had success in predicting odor pleasantness and intensity of human odor perception from chemical features and some semantic descriptors [34, 35]. However, less predictive accuracy was shown in semantic descriptors, and one reason for this is that these descriptors have inherent limits [15, 16, 35]. Some researchers used alternative odor perception data such as odor similarity [36], but still had defects in representing the multidimensional axis of odor quality. Indeed, our method also has the issue of subjective rating but it could to some extent, decrease individual alteration of odor quality. Our study implies that people can measure multidimensional axis of odor quality while reducing individual variations.

Notably, this study did not test all the odors. Therefore, we cannot anticipate that all the odors have the same pattern as IVA in our study. However, it is clear from this study that verbal cues provide higher alteration in LD than UD in the IVA odor profile (Fig 4). Previous studies suggested that primary odor quality exists. Based on this evidence, we hypothesized that primary odor quality may be less affected by top-down modulation such as the verbal cue. In this study, a major alteration was observed in LD rather than in UD, which contains verbal cue related odor quality descriptors (S4 Table), and this support our hypothesis. The reasons we chose IVA are that IVA has already been tested in previous studies and characterized as an ambiguous odor, which can be influenced by verbal cues [19, 27]. Moreover, verbal cue can modulate cingulate cortex and OFC in the IVA condition and this suggests that IVA with the verbal cue can also be changed neurologically [27]. Thus, IVA is one of the odors that possess easily alterable odor quality and similar effects could be expected in less alterable odors. Another notable issue is the possibility that LD may include more random variations than UD and the alteration of LD may arise from random variations. LD includes descriptors less associated with an odor itself and may include more random variations than UD, which may be a major factor. We found that verbal cue affected LD strongly in the second experiment as well (S4 Fig). Moreover, correlations between the first and second experiment data showed an approximately 0.7 r-value in LD, thus verbal cue appeared to have a strong influence on LD descriptors. Although we cannot exclude the possibility of random variations in LD, we here conclude that verbal cue may be the major factor of LD alteration.

Our study assessed the differences in patterns between the cue and no cue conditions in response to the same odor stimulation. We found that high ranked odor quality (UD) may be less affected by top down-modulation (verbal cue) whereas low ranked odor quality (LD) was largely affected compared to the high ranked odor quality. These findings can be applied in research and industry fields, which focus on the quantification of odor quality. Our study suggests that people can extract essential odor quality descriptors, which more precisely represents the odor by sorting data as UD for a better odor quantification. Moreover, people also provide evidence as to why descriptors are rated differently among chemicals. We found that less representative descriptors were significantly influenced by top down-modulation, such as verbal cues; therefore, the rating variation can be different based on the chemicals used. Although the limited numbers of odors tested in this study make it difficult to generalize most odors, our study provides a novel measurement method of multidimensional axis of odor quality with increased accuracy.

## Supporting information

S1 Fig Odor identification. Odor identification of total participants in each group. Y-axis represents a summation of participants’ number which provided from odor identification task (S2 Table). The X-axis represents semantic descriptors that participants used. ‘Parmesan cheese’, ‘cheddar cheese’, ‘blue cheese’ regarded as ‘Cheesy’. ‘Vomit’, ‘stinky foot’, ‘sweat’ regarded as ‘Stinky’. B-IVA had a higher value on ‘Stinky’ compare to ‘Cheesy’, C-IVA had highest value on ‘Cheesy’ and V-IVA had highest value on ‘Stinky’. Most frequently semantic descriptor that provided by participants is ‘vomit’ in B-IVA (vomit: 10, stinky foot: 5, sweat: 8), ‘cheese’ in C-IVA (cheese: 15, parmesan cheese: 3, cheddar cheese: 3), and ‘vomit’ in V-IVA (vomit: 13, stinky foot: 4, sweat: 3).
(TIF)

S2 Fig Statistical patterns of odor descriptors from the second experiment. (A-B) Verification of differences in stimulation conditions. (A) By two-way ANOVA, B-Hep was significantly different from B-IVA but C-IVA and V-IVA had no differences. (B) By two-way ANOVA, C-IVA and B-Hep were significantly different from B-IVA. (C-D) Odor quality space comprised PC1 (C: 34%, D: 21%) and PC2 (C: 8%, D: 11%). Each dot was projected from each participant’s 37 descriptor values in (C) and 109 descriptor values in (D). (E-F) Verification of similarity between stimulation conditions by cluster analysis. Y-axis represents dissimilarity.
(TIF)

S3 Fig Standard error of the mean (SEM) of the correlation coefficient by trials. X-axis is the trial. Y-axis is standard error of the mean. SEM has been shown to converge in over 50 tests **(represented by the red line)**.
(TIF)

S4 Fig Histogram of correlation analysis results between first and second experiments. Orange is comparing real data and blue is comparing shuffled data of the first experiment. The X-axis is the correlation coefficient (r value). Y-axis is the sum of the number of trials. A. Correlation results of first and second experiment data in UD. Most of the trials showed over 0.85 to 0.90 r value. B. Correlation results of first and second experiment data in LD. Most of the trials showed over 0.65 to 0.75 r value.
(TIF)

S1 Table 146 questionnaires for Odor quality rating test. (DOCX)

S2 Table Odor identification task and questionnaires of additional odor responses. (DOCX)

S3 Table Comparison of odor responses between B-IVA versus other stimulation conditions. Each stimulation conditions are compared with B-IVA odor response values.
(DOCX)

S4 Table Descending order of B-IVA odor descriptors. (DOCX)

S1 Dataset Dataset of our studies. This dataset includes odor responses of total experimental conditions (1^st^ and 2^nd^ experiments results of B-IVA, C-IVA, V-IVA and B-Hep).
(XLSX)
