# Supplementary figures and images for: Odor quality profile is partially influenced by verbal cues
Source: PLoS One. 2019 Dec 12;14(12):e0226385. doi: 10.1371/journal.pone.0226385 (PMC6907808; doi:10.1371/journal.pone.0226385)

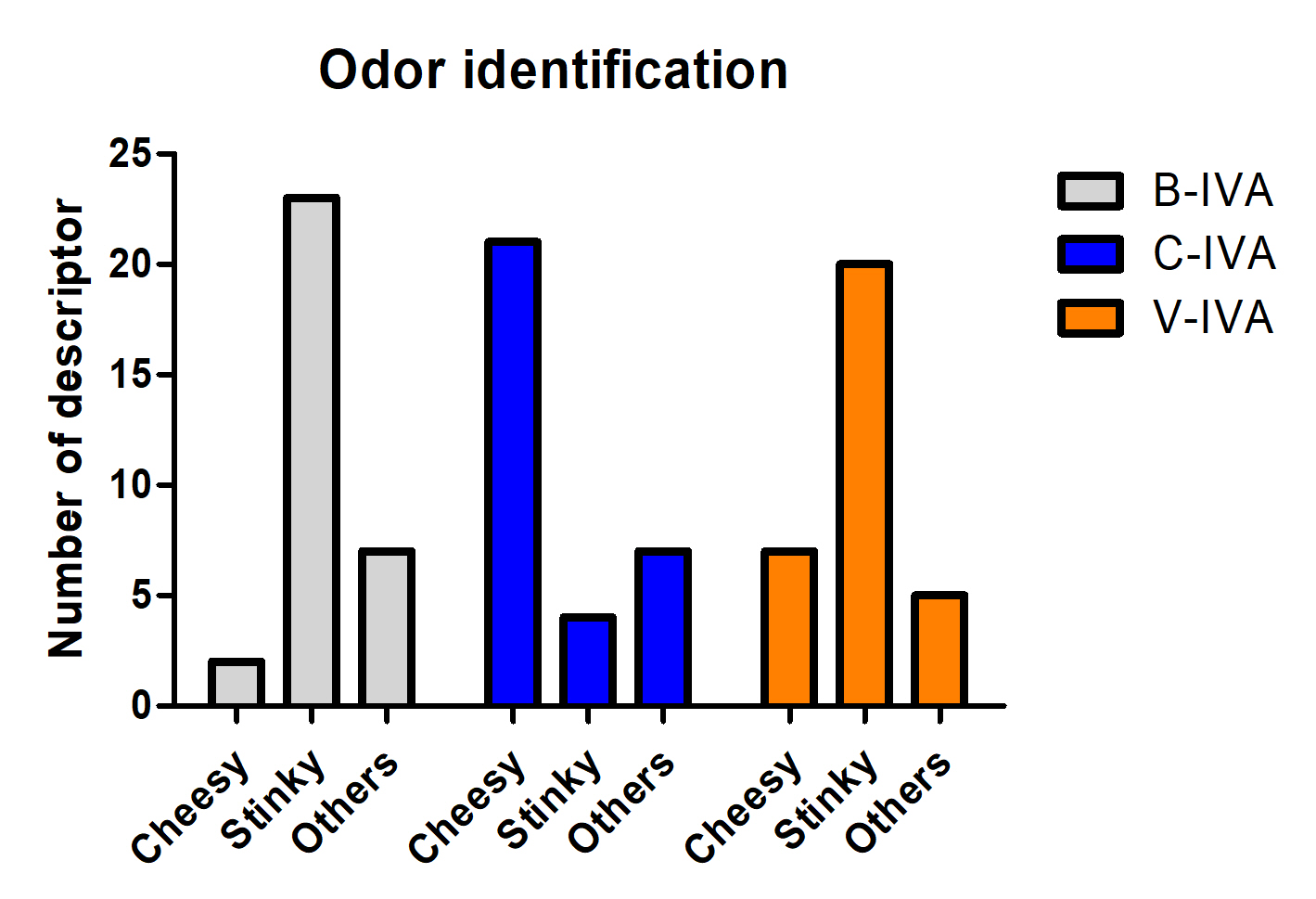

Supplement: S1 Fig — Odor identification of total participants in each group. Y-axis represents a summation of participants’ number which provided from odor identification task (S2 Table). The X-axis represents semantic descriptors that participants used. ‘Parmesan cheese’, ‘cheddar cheese’, ‘blue cheese’ regarded as ‘Cheesy’. ‘Vomit’, ‘stinky foot’, ‘sweat’ regarded as ‘Stinky’. B-IVA had a higher value on ‘Stinky’ compare to ‘Cheesy’, C-IVA had highest value on ‘Cheesy’ and V-IVA had highest value on ‘Stinky’. Most frequently semantic descriptor that provided by participants is ‘vomit’ in B-IVA (vomit: 10, stinky foot: 5, sweat: 8), ‘cheese’ in C-IVA (cheese: 15, parmesan cheese: 3, cheddar cheese: 3), and ‘vomit’ in V-IVA (vomit: 13, stinky foot: 4, sweat: 3). (TIF) [file pone.0226385.s001.tif]

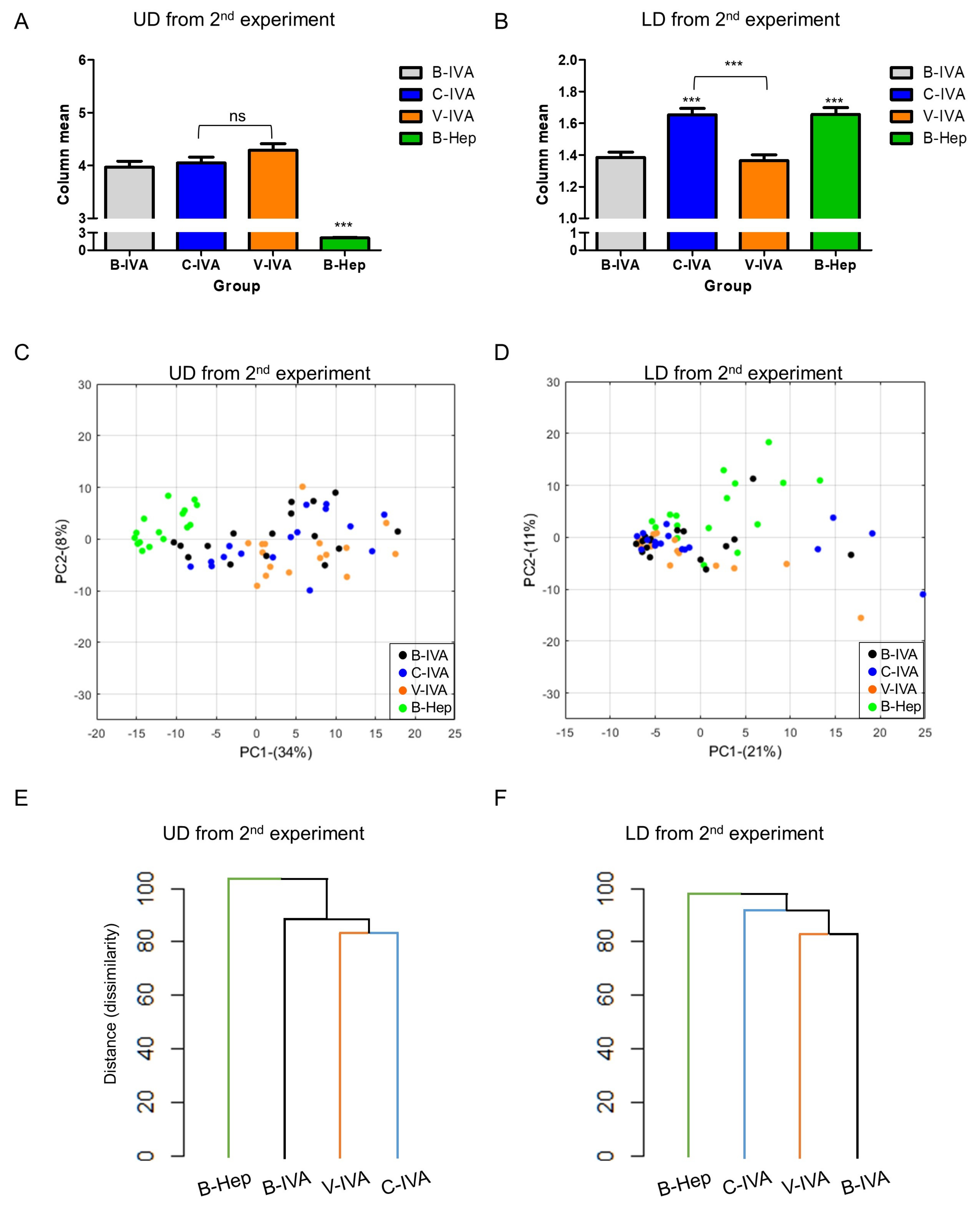

Supplement: S2 Fig — (A-B) Verification of differences in stimulation conditions. (A) By two-way ANOVA, B-Hep was significantly different from B-IVA but C-IVA and V-IVA had no differences. (B) By two-way ANOVA, C-IVA and B-Hep were significantly different from B-IVA. (C-D) Odor quality space comprised PC1 (C: 34%, D: 21%) and PC2 (C: 8%, D: 11%). Each dot was projected from each participant’s 37 descriptor values in (C) and 109 descriptor values in (D). (E-F) Verification of similarity between stimulation conditions by cluster analysis. Y-axis represents dissimilarity. (TIF) [file pone.0226385.s002.tif]

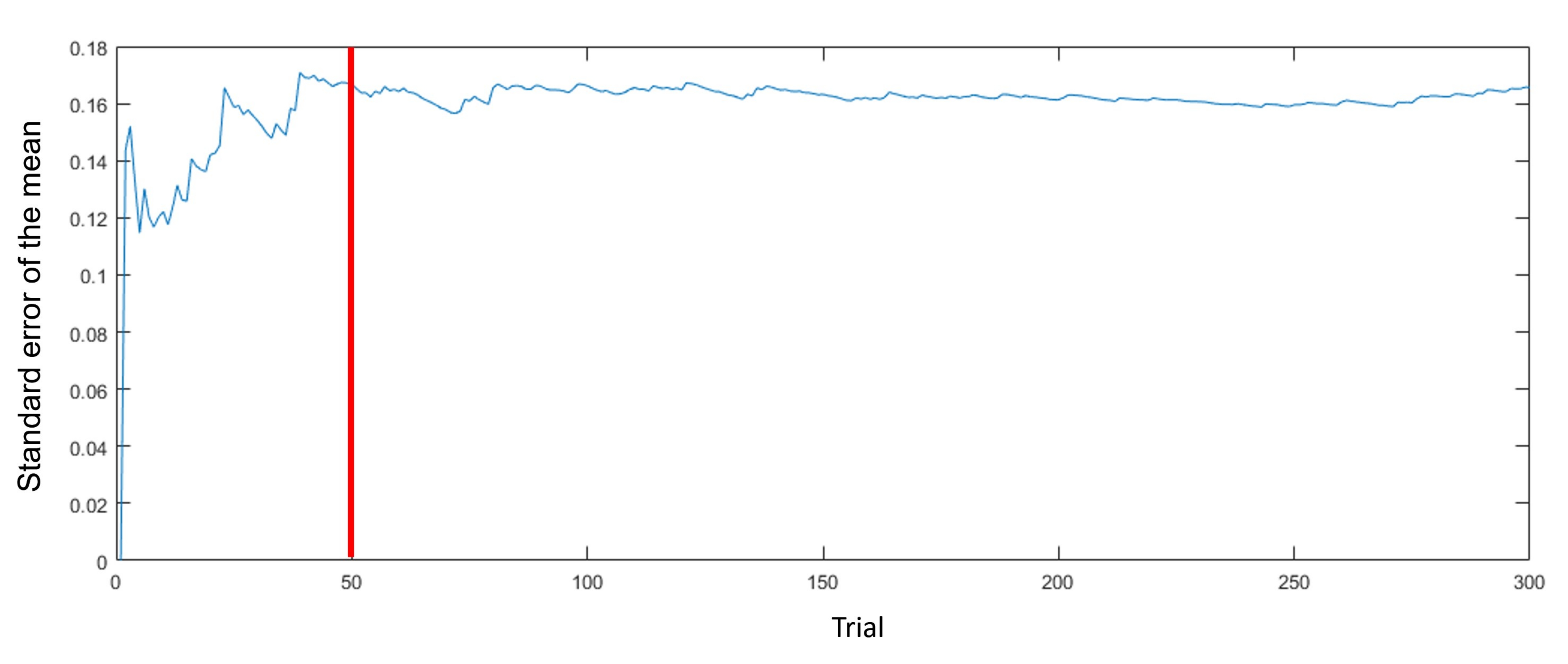

Supplement: S3 Fig — X-axis is the trial. Y-axis is standard error of the mean. SEM has been shown to converge in over 50 tests (represented by the red line). (TIF) [file pone.0226385.s003.tif]

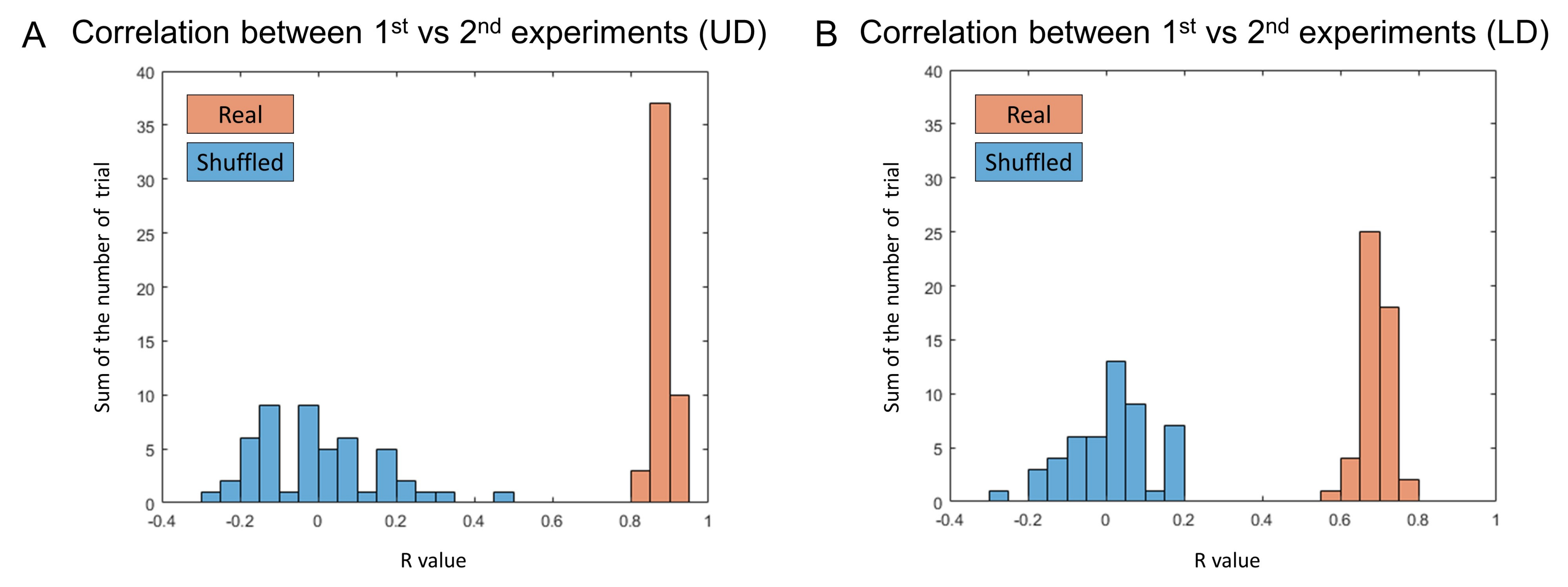

Supplement: S4 Fig — Orange is comparing real data and blue is comparing shuffled data of the first experiment. The X-axis is the correlation coefficient (r value). Y-axis is the sum of the number of trials. A. Correlation results of first and second experiment data in UD. Most of the trials showed over 0.85 to 0.90 r value. B. Correlation results of first and second experiment data in LD. Most of the trials showed over 0.65 to 0.75 r value. (TIF) [file pone.0226385.s004.tif]
