# Supplementary material for: Odor quality profile is partially influenced by verbal cues
Source: PLoS One. 2019 Dec 12;14(12):e0226385. doi: 10.1371/journal.pone.0226385 (PMC6907808; doi:10.1371/journal.pone.0226385)
Supplement: S1 Table — (DOCX) [file pone.0226385.s005.docx]

S1 Table. 146 questionnaires for Odor quality rating test

| **Descriptor** | **Unknown Descriptor** | **Disagree ……** | | | **Neither agree nor disagree** | | | | | **…… Agree** | | | |
| --- | --- | --- | --- | --- | --- | --- | --- | --- | --- | --- | --- | --- | --- |
| alcohol-like | 0 | 1 | 2 | 3 | | 4 | 5 | 6 | 7 | | 8 | 9 |  |
| almond-like | 0 | 1 | 2 | 3 | | 4 | 5 | 6 | 7 | | 8 | 9 |  |
| animal | 0 | 1 | 2 | 3 | | 4 | 5 | 6 | 7 | | 8 | 9 |  |
| anise (licorice) | 0 | 1 | 2 | 3 | | 4 | 5 | 6 | 7 | | 8 | 9 |  |
| apple (fruit) | 0 | 1 | 2 | 3 | | 4 | 5 | 6 | 7 | | 8 | 9 |  |
| aromatic | 0 | 1 | 2 | 3 | | 4 | 5 | 6 | 7 | | 8 | 9 |  |
| bakery (fresh bread) | 0 | 1 | 2 | 3 | | 4 | 5 | 6 | 7 | | 8 | 9 |  |
| banana-like | 0 | 1 | 2 | 3 | | 4 | 5 | 6 | 7 | | 8 | 9 |  |
| bark-like, birch bark | 0 | 1 | 2 | 3 | | 4 | 5 | 6 | 7 | | 8 | 9 |  |
| bean-like | 0 | 1 | 2 | 3 | | 4 | 5 | 6 | 7 | | 8 | 9 |  |
| beery (beer-like) | 0 | 1 | 2 | 3 | | 4 | 5 | 6 | 7 | | 8 | 9 |  |
| bitter | 0 | 1 | 2 | 3 | | 4 | 5 | 6 | 7 | | 8 | 9 |  |
| black pepper-like | 0 | 1 | 2 | 3 | | 4 | 5 | 6 | 7 | | 8 | 9 |  |
| burnt candle | 0 | 1 | 2 | 3 | | 4 | 5 | 6 | 7 | | 8 | 9 |  |
| burnt milk | 0 | 1 | 2 | 3 | | 4 | 5 | 6 | 7 | | 8 | 9 |  |
| burnt rubber-like | 0 | 1 | 2 | 3 | | 4 | 5 | 6 | 7 | | 8 | 9 |  |
| burnt, smoky | 0 | 1 | 2 | 3 | | 4 | 5 | 6 | 7 | | 8 | 9 |  |
| buttery (fresh) | 0 | 1 | 2 | 3 | | 4 | 5 | 6 | 7 | | 8 | 9 |  |
| cadaverous, like dead animal | 0 | 1 | 2 | 3 | | 4 | 5 | 6 | 7 | | 8 | 9 |  |
| camphor-like | 0 | 1 | 2 | 3 | | 4 | 5 | 6 | 7 | | 8 | 9 |  |
| cantaloupe, honey dew melon | 0 | 1 | 2 | 3 | | 4 | 5 | 6 | 7 | | 8 | 9 |  |
| caramel | 0 | 1 | 2 | 3 | | 4 | 5 | 6 | 7 | | 8 | 9 |  |
| caraway | 0 | 1 | 2 | 3 | | 4 | 5 | 6 | 7 | | 8 | 9 |  |
| cardboard-like | 0 | 1 | 2 | 3 | | 4 | 5 | 6 | 7 | | 8 | 9 |  |
| cat-urine-like | 0 | 1 | 2 | 3 | | 4 | 5 | 6 | 7 | | 8 | 9 |  |
| cedarwood-like | 0 | 1 | 2 | 3 | | 4 | 5 | 6 | 7 | | 8 | 9 |  |
| celery | 0 | 1 | 2 | 3 | | 4 | 5 | 6 | 7 | | 8 | 9 |  |
| chalky | 0 | 1 | 2 | 3 | | 4 | 5 | 6 | 7 | | 8 | 9 |  |
| cheesy | 0 | 1 | 2 | 3 | | 4 | 5 | 6 | 7 | | 8 | 9 |  |
| chemical | 0 | 1 | 2 | 3 | | 4 | 5 | 6 | 7 | | 8 | 9 |  |
| cherry (berry) | 0 | 1 | 2 | 3 | | 4 | 5 | 6 | 7 | | 8 | 9 |  |
| chocolate | 0 | 1 | 2 | 3 | | 4 | 5 | 6 | 7 | | 8 | 9 |  |
| cinnamon | 0 | 1 | 2 | 3 | | 4 | 5 | 6 | 7 | | 8 | 9 |  |
| clove-like | 0 | 1 | 2 | 3 | | 4 | 5 | 6 | 7 | | 8 | 9 |  |
| coconut-like | 0 | 1 | 2 | 3 | | 4 | 5 | 6 | 7 | | 8 | 9 |  |
| coffee-like | 0 | 1 | 2 | 3 | | 4 | 5 | 6 | 7 | | 8 | 9 |  |
| cologne | 0 | 1 | 2 | 3 | | 4 | 5 | 6 | 7 | | 8 | 9 |  |
| cooked vegetables | 0 | 1 | 2 | 3 | | 4 | 5 | 6 | 7 | | 8 | 9 |  |
| cool, cooling | 0 | 1 | 2 | 3 | | 4 | 5 | 6 | 7 | | 8 | 9 |  |
| cork-like | 0 | 1 | 2 | 3 | | 4 | 5 | 6 | 7 | | 8 | 9 |  |
| creosote | 0 | 1 | 2 | 3 | | 4 | 5 | 6 | 7 | | 8 | 9 |  |
| crushed-grass | 0 | 1 | 2 | 3 | | 4 | 5 | 6 | 7 | | 8 | 9 |  |
| crushed-weeds | 0 | 1 | 2 | 3 | | 4 | 5 | 6 | 7 | | 8 | 9 |  |
| dill-like | 0 | 1 | 2 | 3 | | 4 | 5 | 6 | 7 | | 8 | 9 |  |
| dirty linen-like | 0 | 1 | 2 | 3 | | 4 | 5 | 6 | 7 | | 8 | 9 |  |
| disinfectant, carbolic | 0 | 1 | 2 | 3 | | 4 | 5 | 6 | 7 | | 8 | 9 |  |
| dry, powdery | 0 | 1 | 2 | 3 | | 4 | 5 | 6 | 7 | | 8 | 9 |  |
| eggy (fresh eggs) | 0 | 1 | 2 | 3 | | 4 | 5 | 6 | 7 | | 8 | 9 |  |
| etherish, anaesthetic | 0 | 1 | 2 | 3 | | 4 | 5 | 6 | 7 | | 8 | 9 |  |
| eucalyptus | 0 | 1 | 2 | 3 | | 4 | 5 | 6 | 7 | | 8 | 9 |  |
| fecal (like manure) | 0 | 1 | 2 | 3 | | 4 | 5 | 6 | 7 | | 8 | 9 |  |
| fermented (rotten) fruit | 0 | 1 | 2 | 3 | | 4 | 5 | 6 | 7 | | 8 | 9 |  |
| fishy | 0 | 1 | 2 | 3 | | 4 | 5 | 6 | 7 | | 8 | 9 |  |
| floral | 0 | 1 | 2 | 3 | | 4 | 5 | 6 | 7 | | 8 | 9 |  |
| fragrant | 0 | 1 | 2 | 3 | | 4 | 5 | 6 | 7 | | 8 | 9 |  |
| fresh green vegetables | 0 | 1 | 2 | 3 | | 4 | 5 | 6 | 7 | | 8 | 9 |  |
| fresh tobacco smoke | 0 | 1 | 2 | 3 | | 4 | 5 | 6 | 7 | | 8 | 9 |  |
| fried chicken | 0 | 1 | 2 | 3 | | 4 | 5 | 6 | 7 | | 8 | 9 |  |
| fruity (citrus) | 0 | 1 | 2 | 3 | | 4 | 5 | 6 | 7 | | 8 | 9 |  |
| fruity (other) | 0 | 1 | 2 | 3 | | 4 | 5 | 6 | 7 | | 8 | 9 |  |
| garlic, onion | 0 | 1 | 2 | 3 | | 4 | 5 | 6 | 7 | | 8 | 9 |  |
| geranium leaves | 0 | 1 | 2 | 3 | | 4 | 5 | 6 | 7 | | 8 | 9 |  |
| grainy (as in grain) | 0 | 1 | 2 | 3 | | 4 | 5 | 6 | 7 | | 8 | 9 |  |
| grape-juice-like | 0 | 1 | 2 | 3 | | 4 | 5 | 6 | 7 | | 8 | 9 |  |
| grapefruit | 0 | 1 | 2 | 3 | | 4 | 5 | 6 | 7 | | 8 | 9 |  |
| green pepper | 0 | 1 | 2 | 3 | | 4 | 5 | 6 | 7 | | 8 | 9 |  |
| hay | 0 | 1 | 2 | 3 | | 4 | 5 | 6 | 7 | | 8 | 9 |  |
| heavy | 0 | 1 | 2 | 3 | | 4 | 5 | 6 | 7 | | 8 | 9 |  |
| herbal, green, cut grass | 0 | 1 | 2 | 3 | | 4 | 5 | 6 | 7 | | 8 | 9 |  |
| honey-like | 0 | 1 | 2 | 3 | | 4 | 5 | 6 | 7 | | 8 | 9 |  |
| household gas | 0 | 1 | 2 | 3 | | 4 | 5 | 6 | 7 | | 8 | 9 |  |
| incense | 0 | 1 | 2 | 3 | | 4 | 5 | 6 | 7 | | 8 | 9 |  |
| kerosene | 0 | 1 | 2 | 3 | | 4 | 5 | 6 | 7 | | 8 | 9 |  |
| kippery (smoked fish) | 0 | 1 | 2 | 3 | | 4 | 5 | 6 | 7 | | 8 | 9 |  |
| laurel leaves | 0 | 1 | 2 | 3 | | 4 | 5 | 6 | 7 | | 8 | 9 |  |
| lavender | 0 | 1 | 2 | 3 | | 4 | 5 | 6 | 7 | | 8 | 9 |  |
| leather-like | 0 | 1 | 2 | 3 | | 4 | 5 | 6 | 7 | | 8 | 9 |  |
| lemon (fruit) | 0 | 1 | 2 | 3 | | 4 | 5 | 6 | 7 | | 8 | 9 |  |
| light | 0 | 1 | 2 | 3 | | 4 | 5 | 6 | 7 | | 8 | 9 |  |
| like ammonia | 0 | 1 | 2 | 3 | | 4 | 5 | 6 | 7 | | 8 | 9 |  |
| like blood, raw meat | 0 | 1 | 2 | 3 | | 4 | 5 | 6 | 7 | | 8 | 9 |  |
| like burnt paper | 0 | 1 | 2 | 3 | | 4 | 5 | 6 | 7 | | 8 | 9 |  |
| like cleaning fluid (carbona) | 0 | 1 | 2 | 3 | | 4 | 5 | 6 | 7 | | 8 | 9 |  |
| like gasoline, solvent | 0 | 1 | 2 | 3 | | 4 | 5 | 6 | 7 | | 8 | 9 |  |
| like mothballs | 0 | 1 | 2 | 3 | | 4 | 5 | 6 | 7 | | 8 | 9 |  |
| malty | 0 | 1 | 2 | 3 | | 4 | 5 | 6 | 7 | | 8 | 9 |  |
| maple (as in syrup) | 0 | 1 | 2 | 3 | | 4 | 5 | 6 | 7 | | 8 | 9 |  |
| meaty (cooked, good) | 0 | 1 | 2 | 3 | | 4 | 5 | 6 | 7 | | 8 | 9 |  |
| medicinal | 0 | 1 | 2 | 3 | | 4 | 5 | 6 | 7 | | 8 | 9 |  |
| metallic | 0 | 1 | 2 | 3 | | 4 | 5 | 6 | 7 | | 8 | 9 |  |
| minty, peppermint | 0 | 1 | 2 | 3 | | 4 | 5 | 6 | 7 | | 8 | 9 |  |
| molasses | 0 | 1 | 2 | 3 | | 4 | 5 | 6 | 7 | | 8 | 9 |  |
| mouse-like | 0 | 1 | 2 | 3 | | 4 | 5 | 6 | 7 | | 8 | 9 |  |
| mushroom-like | 0 | 1 | 2 | 3 | | 4 | 5 | 6 | 7 | | 8 | 9 |  |
| musk-like | 0 | 1 | 2 | 3 | | 4 | 5 | 6 | 7 | | 8 | 9 |  |
| musty, earthy, moldy | 0 | 1 | 2 | 3 | | 4 | 5 | 6 | 7 | | 8 | 9 |  |
| nail polish remover | 0 | 1 | 2 | 3 | | 4 | 5 | 6 | 7 | | 8 | 9 |  |
| nutty (walnut, etc.) | 0 | 1 | 2 | 3 | | 4 | 5 | 6 | 7 | | 8 | 9 |  |
| oak wood, cognac-like | 0 | 1 | 2 | 3 | | 4 | 5 | 6 | 7 | | 8 | 9 |  |
| oily, fatty | 0 | 1 | 2 | 3 | | 4 | 5 | 6 | 7 | | 8 | 9 |  |
| orange (fruit) | 0 | 1 | 2 | 3 | | 4 | 5 | 6 | 7 | | 8 | 9 |  |
| paint-like | 0 | 1 | 2 | 3 | | 4 | 5 | 6 | 7 | | 8 | 9 |  |
| peach (fruit) | 0 | 1 | 2 | 3 | | 4 | 5 | 6 | 7 | | 8 | 9 |  |
| peanut butter | 0 | 1 | 2 | 3 | | 4 | 5 | 6 | 7 | | 8 | 9 |  |
| pear (fruit) | 0 | 1 | 2 | 3 | | 4 | 5 | 6 | 7 | | 8 | 9 |  |
| perfumery | 0 | 1 | 2 | 3 | | 4 | 5 | 6 | 7 | | 8 | 9 |  |
| pineapple (fruit) | 0 | 1 | 2 | 3 | | 4 | 5 | 6 | 7 | | 8 | 9 |  |
| popcorn | 0 | 1 | 2 | 3 | | 4 | 5 | 6 | 7 | | 8 | 9 |  |
| putrid, foul, decayed | 0 | 1 | 2 | 3 | | 4 | 5 | 6 | 7 | | 8 | 9 |  |
| raisins | 0 | 1 | 2 | 3 | | 4 | 5 | 6 | 7 | | 8 | 9 |  |
| rancid | 0 | 1 | 2 | 3 | | 4 | 5 | 6 | 7 | | 8 | 9 |  |
| raw cucumber-like | 0 | 1 | 2 | 3 | | 4 | 5 | 6 | 7 | | 8 | 9 |  |
| raw potato-like | 0 | 1 | 2 | 3 | | 4 | 5 | 6 | 7 | | 8 | 9 |  |
| rope-like | 0 | 1 | 2 | 3 | | 4 | 5 | 6 | 7 | | 8 | 9 |  |
| rose-like | 0 | 1 | 2 | 3 | | 4 | 5 | 6 | 7 | | 8 | 9 |  |
| rubbery (new rubber) | 0 | 1 | 2 | 3 | | 4 | 5 | 6 | 7 | | 8 | 9 |  |
| sauerkraut-like | 0 | 1 | 2 | 3 | | 4 | 5 | 6 | 7 | | 8 | 9 |  |
| seasoning (for meat) | 0 | 1 | 2 | 3 | | 4 | 5 | 6 | 7 | | 8 | 9 |  |
| seminal, sperm-like | 0 | 1 | 2 | 3 | | 4 | 5 | 6 | 7 | | 8 | 9 |  |
| sewer odor | 0 | 1 | 2 | 3 | | 4 | 5 | 6 | 7 | | 8 | 9 |  |
| sharp, pungent, acid | 0 | 1 | 2 | 3 | | 4 | 5 | 6 | 7 | | 8 | 9 |  |
| sickening | 0 | 1 | 2 | 3 | | 4 | 5 | 6 | 7 | | 8 | 9 |  |
| soapy | 0 | 1 | 2 | 3 | | 4 | 5 | 6 | 7 | | 8 | 9 |  |
| sooty | 0 | 1 | 2 | 3 | | 4 | 5 | 6 | 7 | | 8 | 9 |  |
| soupy | 0 | 1 | 2 | 3 | | 4 | 5 | 6 | 7 | | 8 | 9 |  |
| sour milk | 0 | 1 | 2 | 3 | | 4 | 5 | 6 | 7 | | 8 | 9 |  |
| sour | 0 | 1 | 2 | 3 | | 4 | 5 | 6 | 7 | | 8 | 9 |  |
| spicy | 0 | 1 | 2 | 3 | | 4 | 5 | 6 | 7 | | 8 | 9 |  |
| stale | 0 | 1 | 2 | 3 | | 4 | 5 | 6 | 7 | | 8 | 9 |  |
| stale tobacco smoke | 0 | 1 | 2 | 3 | | 4 | 5 | 6 | 7 | | 8 | 9 |  |
| strawberry-like | 0 | 1 | 2 | 3 | | 4 | 5 | 6 | 7 | | 8 | 9 |  |
| sulphidic | 0 | 1 | 2 | 3 | | 4 | 5 | 6 | 7 | | 8 | 9 |  |
| Sweaty | 0 | 1 | 2 | 3 | | 4 | 5 | 6 | 7 | | 8 | 9 |  |
| sweet | 0 | 1 | 2 | 3 | | 4 | 5 | 6 | 7 | | 8 | 9 |  |
| tar-like | 0 | 1 | 2 | 3 | | 4 | 5 | 6 | 7 | | 8 | 9 |  |
| tea-leaves-like | 0 | 1 | 2 | 3 | | 4 | 5 | 6 | 7 | | 8 | 9 |  |
| turpentine (pine oil) | 0 | 1 | 2 | 3 | | 4 | 5 | 6 | 7 | | 8 | 9 |  |
| urine-like | 0 | 1 | 2 | 3 | | 4 | 5 | 6 | 7 | | 8 | 9 |  |
| vanilla-like | 0 | 1 | 2 | 3 | | 4 | 5 | 6 | 7 | | 8 | 9 |  |
| varnish | 0 | 1 | 2 | 3 | | 4 | 5 | 6 | 7 | | 8 | 9 |  |
| violets | 0 | 1 | 2 | 3 | | 4 | 5 | 6 | 7 | | 8 | 9 |  |
| warm | 0 | 1 | 2 | 3 | | 4 | 5 | 6 | 7 | | 8 | 9 |  |
| wet paper-like | 0 | 1 | 2 | 3 | | 4 | 5 | 6 | 7 | | 8 | 9 |  |
| wet wool, wet dog | 0 | 1 | 2 | 3 | | 4 | 5 | 6 | 7 | | 8 | 9 |  |
| woody, resinous | 0 | 1 | 2 | 3 | | 4 | 5 | 6 | 7 | | 8 | 9 |  |
| yeasty | 0 | 1 | 2 | 3 | | 4 | 5 | 6 | 7 | | 8 | 9 |  |
