# Supplementary material for: Odor quality profile is partially influenced by verbal cues
Source: PLoS One. 2019 Dec 12;14(12):e0226385. doi: 10.1371/journal.pone.0226385 (PMC6907808; doi:10.1371/journal.pone.0226385)
Supplement: S2 Table — (DOCX) [file pone.0226385.s006.docx]

S2 Table. Odor identification task and questionnaires of additional odor responses

| Identify the odor |  | | | | | | | | |
| --- | --- | --- | --- | --- | --- | --- | --- | --- | --- |
|  |  |  |  |  |  |  |  |  |  |
|  | **Low** | | | **…………………………** | | | **High** | | |
| Pleasantness | 1 | 2 | 3 | 4 | 5 | 6 | 7 | 8 | 9 |
| Intensity | 1 | 2 | 3 | 4 | 5 | 6 | 7 | 8 | 9 |
| Familiarity | 1 | 2 | 3 | 4 | 5 | 6 | 7 | 8 | 9 |
| Edibility | 1 | 2 | 3 | 4 | 5 | 6 | 7 | 8 | 9 |
| Relaxing effect | 1 | 2 | 3 | 4 | 5 | 6 | 7 | 8 | 9 |
