# Supplementary material for: Odor quality profile is partially influenced by verbal cues
Source: PLoS One. 2019 Dec 12;14(12):e0226385. doi: 10.1371/journal.pone.0226385 (PMC6907808; doi:10.1371/journal.pone.0226385)
Supplement: S3 Table — Each stimulation conditions are compared with B-IVA odor response values. (DOCX) [file pone.0226385.s007.docx]

S3 Table. Comparison of odor responses between B-IVA versus other stimulation conditions. Each stimulation conditions are compared with B-IVA odor response values.

|  | B-IVA vs C-IVA | | B-IVA vs V-IVA | | B-IVA vs B-Hep | |
| --- | --- | --- | --- | --- | --- | --- |
|  | ***p-value*** | ***t-value*** | ***p-value*** | ***t-value*** | ***p-value*** | ***t-value*** |
| Pleasantness | <0.001 (***) | 3.54 | 0.72 | 0.35 | <0.001 (***) | 9.83 |
| Intensity | 0.94 | 0.078 | 0.25 | 1.17 | 0.067 | -1.86 |
| Familiarity | 0.26 | 1.14 | 0.55 | 0.60 | 0.22 | 1.23 |
| Edibility | <0.001 (***) | 5.081 | 0.23 | 1.21 | <0.001 (***) | 4.54 |
| Relaxing effect | 0.38 | 0.88 | 0.12 | -1.57 | <0.001 (***) | 6.43 |

***two tail t-test, DF=62 * (0.05<p), ** (0.01<p), *** (0.001<p)***
