# Supplementary material for: Odor quality profile is partially influenced by verbal cues
Source: PLoS One. 2019 Dec 12;14(12):e0226385. doi: 10.1371/journal.pone.0226385 (PMC6907808; doi:10.1371/journal.pone.0226385)
Supplement: S4 Table — (DOCX) [file pone.0226385.s008.docx]

**S4 Table. Descending order of B-IVA odor descriptors**

| 1 | sickening | 31 | medicinal | 61 | cooked vegetables | 91 | strawberry-like | 121 | cedarwood-like |
| --- | --- | --- | --- | --- | --- | --- | --- | --- | --- |
| 2 | aromatic | 32 | wet wool, wet dog | 62 | chalky | 92 | kerosene | 122 | lavender |
| 3 | stale | 33 | oily, fatty | 63 | honey-like | 93 | spicy | 123 | varnish |
| 4 | dirty linen-like | 34 | rubbery (new rubber) | 64 | burnt rubber-like | 94 | peach (fruit) | 124 | green pepper |
| 5 | rancid | 35 | sulphidic | 65 | sauerkraut-like | 95 | crushed-weeds | 125 | cherry (berry) |
| 6 | Sweaty | 36 | fishy | 66 | tar-like | 96 | grapefruit | 126 | fried chicken |
| 7 | putrid, foul, decayed | 37 | cork-like | 67 | almond-like | 97 | disinfectant, carbolic | 127 | burnt milk |
| 8 | musty, earthy, moldy | 38 | banana-like | 68 | beery (beer-like) | 98 | bean-like | 128 | oak wood, cognac-like |
| 9 | like ammonia | 39 | floral | 69 | sooty | 99 | burnt candle | 129 | raw cucumber-like |
| 10 | fecal (like manure) | 40 | raisins | 70 | paint-like | 100 | soupy | 130 | minty, peppermint |
| 11 | heavy | 41 | fruity (other) | 71 | rose-like | 101 | black pepper-like | 131 | fresh green vegetables |
| 12 | animal | 42 | turpentine (pine oil) | 72 | crushed-grass | 102 | meaty (cooked, good) | 132 | celery |
| 13 | sewer odor | 43 | metallic | 73 | bakery (fresh bread) | 103 | alcohol-like | 133 | malty |
| 14 | urine-like | 44 | woody, resinous | 74 | soapy | 104 | like gasoline, solvent | 134 | dry, powdery |
| 15 | fermented (rotten) fruit | 45 | sweet | 75 | lemon (fruit) | 105 | apple (fruit) | 135 | violets |
| 16 | sour | 46 | garlic, onion | 76 | household gas | 106 | cologne | 136 | molasses |
| 17 | cheesy | 47 | hay | 77 | cool, cooling | 107 | caramel | 137 | eucalyptus |
| 18 | light | 48 | raw potato-like | 78 | herbal, green, cut grass | 108 | perfumery | 138 | musk-like |
| 19 | sour milk | 49 | coconut-like | 79 | grape-juice-like | 109 | coffee-like | 139 | etherish, anaesthetic |
| 20 | cadaverous, like dead animal | 50 | fruity (citrus) | 80 | tea-leaves-like | 110 | pear (fruit) | 140 | creosote |
| 21 | cat-urine-like | 51 | like mothballs | 81 | kippery (smoked fish) | 111 | cantaloupe, honey dew melon | 141 | laurel leaves |
| 22 | leather-like | 52 | seasoning (for meat) | 82 | orange (fruit) | 112 | cinnamon | 142 | dill-like |
| 23 | warm | 53 | wet paper-like | 83 | eggy (fresh eggs) | 113 | popcorn | 143 | caraway |
| 24 | chemical | 54 | bark-like, birch bark | 84 | buttery (fresh) | 114 | seminal, sperm-like | 144 | camphor-like |
| 25 | mouse-like | 55 | grainy (as in grain) | 85 | vanilla-like | 115 | nail polish remover | 145 | geranium leaves |
| 26 | sharp, pungent, acid | 56 | nutty (walnut, etc.) | 86 | peanut butter | 116 | fragrant | 146 | incense |
| 27 | mushroom-like | 57 | stale tobacco smoke | 87 | cardboard-like | 117 | like burnt paper | - | - |
| 28 | bitter | 58 | pineapple (fruit) | 88 | fresh tobacco smoke | 118 | clove-like | - | - |
| 29 | yeasty | 59 | like cleaning fluid (carbona) | 89 | anise (licorice) | 119 | maple (as in syrup) | - | - |
| 30 | rope-like | 60 | like blood, raw meat | 90 | burnt, smoky | 120 | chocolate | - | - |
